# The ICaRAS randomised controlled trial: Intravenous iron to treat anaemia in people with advanced cancer – feasibility of recruitment, intervention and delivery

**DOI:** 10.1177/02692163221145604

**Published:** 2023-01-06

**Authors:** Edward A Dickson, Oliver Ng, Barrie D Keeler, Andrew Wilcock, Matthew J Brookes, Austin G Acheson

**Affiliations:** 1 National Institute for Health Research Biomedical Research Centre in Gastrointestinal and Liver Diseases, Nottingham University Hospitals NHS Trust, Nottingham, UK; 2 Department of Colorectal Surgery, Nottingham University Hospitals NHS Trust, University of Nottingham, Nottingham, UK; 3 Milton Keynes University Hospitals NHS Foundation Trust, Milton Keynes, UK; 4 The University of Buckingham, Buckingham, MK18 1EG, UK; 5 Department of Palliative Care, University of Nottingham, Nottingham, UK; 6 Department of Gastroenterology, Royal Wolverhampton NHS Trust, Wolverhampton, UK; 7 Faculty of Science and Engineering, University of Wolverhampton, Wolverhampton, UK

**Keywords:** Anaemia, advanced cancer, intravenous iron, randomised controlled trial, fatigue, quality of life

## Abstract

**Background:**

Anaemia is highly prevalent in people with advanced, palliative cancer yet sufficiently effective and safe treatments are lacking. Oral iron is poorly tolerated, and blood transfusion offers only transient benefits. Intravenous iron has shown promise as an effective treatment for anaemia but its use for people with advanced, palliative cancer lacks evidence.

**Aims:**

To assess feasibility of the trial design according to screening, recruitment, and attrition rates. To evaluate the efficacy of intravenous iron to treat anaemia in people with solid tumours, receiving palliative care.

**Design:**

A multicentre, randomised, double blind, placebo-controlled trial of intravenous iron (ferric derisomaltose, Monofer^®^). Outcomes included trial feasibility, change in blood indices, and change in quality of life via three validated questionnaires (EQ5D5L, QLQC30, and the FACIT-F) over 8 weeks. (ISRCTN; 13370767).

**Setting/Participants:**

People with anaemia and advanced solid tumours who were fatigued with a performance status ⩽2 receiving support from a specialist palliative care service.

**Results:**

34 participants were randomised over 16 months (17 iron, 17 placebo). Among those eligible 47% of people agreed to participate and total study attrition was 26%. Blinding was successful in all participants. There were no serious adverse reactions. Results indicated that intravenous iron may be efficacious at improving participant haemoglobin, iron stores and select fatigue specific quality of life measures compared to placebo.

**Conclusion:**

The trial was feasible according to recruitment and attrition rates. Intravenous iron increased haemoglobin and may improve fatigue specific quality of life measures compared to placebo. A definitive trial is required for confirmation.

**What is already known about the topic?**

- Anaemia is highly prevalent in people with palliative solid tumours.
- Existing treatments such as oral iron or blood transfusions are limited by a lack of efficacy or transient benefit.
- Intravenous iron has seen increasing adoption in the management of people with anaemia, but evidence is lacking in the palliative cancer setting.

**What this paper adds**

- It is feasible to administer intravenous iron in this group of people in this setting.
- People were willing and able to be randomised to receive intravenous iron or placebo in this trial.
- An indication that intravenous iron may be efficacious as correcting anaemia and improving quality of life in this group of people.

**Implications for practice, theory, or policy**

- This randomised controlled trial supports the further evaluation of intravenous iron to treat anaemia in people with palliative solid tumours.
- The results signal the need for a definitive trial to further confirm these findings.
- Further evidence may lead to the adoption of intravenous iron as a treatment option for people in this setting.

## Introduction

Anaemia affects over 70% of people with advanced cancer.^1^ Symptoms include fatigue, breathlessness and reduced physical activity, which impact negatively on quality of life.^2^ In the palliative care setting treatments for this anaemia should focus on alleviating these debilitating symptoms. However, sufficiently effective, and safe treatments are lacking. Blood transfusions are a finite and costly resource and carry a risk of significant harm to recipients. They may only offer a temporary benefit, with improvements in fatigue lasting less than 2 weeks for most people.^3,4^ There is controversy regarding the use of erythropoietin in this setting. For people not receiving concurrent chemotherapy erythropoietin may be associated with an increased mortality risk.^5^ However, in people receiving myelosuppressive chemotherapy this association has more recently been refuted.^6^

Oral iron has been a popular treatment, but nausea and gastric irritation are common,^7^ resulting in non-adherence rates of up to 40% among recipients.^8^ Importantly, efficacy is impeaded by a relatively poor oral bio-availability (about 16%–21%)^9^ that is reduced to potentially negligible levels in people with cancer due to the inflammatory upregulation of hepcidin, the key determinant of the availability of absorbed iron.^10,11^

Compared to oral iron, intravenous iron works more rapidly and has been used safely and effectively for the treatment of people with anaemia in colorectal cancer surgery^12^ inflammatory bowel disease,^13^ heart failure,^14^ and chronic kidney disease.^15^ However, there is limited evidence regarding its use for anaemia in people with advanced cancer.^16^ This paper reports the feasibility of recruitment, randomisation, intervention, and participant follow up from the first randomised controlled trial of intravenous iron administration for anaemia in people with advanced cancer – The ICaRAS trial (Intravenous Iron for Cancer Related Anaemia Symptoms). We also report the impact of intravenous iron on the symptomology of this anaemia in trial participants.

## Methods

### Design

We conducted a multicentre, randomised, double blind, placebo-controlled feasibility trial of intravenous iron therapy to treat anaemia in people with advanced cancer suffering from fatigue.

### Research questions

The primary outcome was an assessment of feasibility of the trial design, its inclusion/exclusion criteria, and the proposed intervention. The study was designed to answer the following research questions

- Can we recruit the target sample size during the proposed study duration according to the participant inclusion criteria? (screening and recruitment)
- Are eligible participants willing and able to enrol in the study? (acceptability)
- Does the study duration allow the proposed outcome measures to be evaluated whilst accounting for the impact of participant withdrawal? (attrition)
- Can intravenous iron be administered safely administered in this group of people according to tolerability and adverse events? (safety)
- Can the chosen secondary outcome measures be completed by participants, and can the data obtained from these inform the primary outcome for a definitive trial? (adherence)
- What are the logistical and organisational challenges of delivering this trial in the chosen setting?
- Is there evidence that intravenous iron might be efficacious at improving anaemia related symptoms in these people compared to placebo?

### Population

Anaemic people with histologically or radiologically proven solid epithelial tumours not amenable to curative treatment were recruited. Participant inclusion criteria included anaemia according to the WHO definition (haemoglobin <130 g/L men and <120 g/L women), a fatigue score ⩾4 out of 10 on a linear scale (0 no fatigue, 10 worst fatigue imaginable) and an ECOG performance status ⩽2. People with a haematological malignancy, those who had received intravenous iron in the preceding 3 months, an allogenic blood transfusion in the last 2 weeks, or those with signs of active bleeding were excluded. Those who had undergone chemotherapy and/or immunotherapy and/or radiotherapy within 8 weeks were also excluded due to the confounding myelosuppressive effect of these treatments. Participants taking oral iron were required to stop 1 week before their infusion and for the duration of enrolment. Full inclusion/exclusion criteria are listed in Supplemental Table 1.

### Setting

The study was conducted at two UK secondary care institutions with a specialist palliative care service on site. Both centres offered inpatient and outpatient palliative care alongside a regular day therapy service.

### Recruitment

People referred from oncology or surgical multidisciplinary team (MDT) meetings to the palliative care service were sequentially screened by the research team alongside existing outpatient and day therapy attendees. People were screened at any stage of their involvement with specialist palliative care services. They were informed about the study by their usual palliative care clinician. To recruit the target of 40 participants the study was planned to run for 2 years.

### Randomisation

After recruitment, participants were randomised via an online platform (Sealed Envelope Ltd. 2021) in a 1:1 fashion to receive either an infusion of intravenous iron (Ferric derisomaltose, Monofer^®^ – Pharmacosmos Ltd, Holbaek, Denmark) or placebo (250 ml 0.9% sodium chloride).

### Intervention

Infusions were administered to participants at the palliative care day therapy unit by two unblinded nurse investigators. The difference in the appearance of the iron and placebo was concealed using an opaque intravenous administration set (Intrafix Primeline UV protect, B. Braun Ltd, Melsungen, Germany). Iron was dosed according to the simplified dosing regimen which calculates body iron deficit by weight and baseline haemoglobin (Supplemental Table 2). For individuals exceeding the maximum 20 mg/kg/week dose an infusion of the maximum permitted dose was administered at a first visit with a second dose of the remainder administered 7–10 days later. The same protocol was undertaken for participants in the placebo arm to ensure blinding was maintained. Adherence to the administration protocol, participant monitoring via 15 min observations during and after the infusion, and maintenance of successful blinding were all recorded. Participant follow up was conducted at 4 and 8 weeks after infusion.

### Secondary outcomes

**Figure 1.**
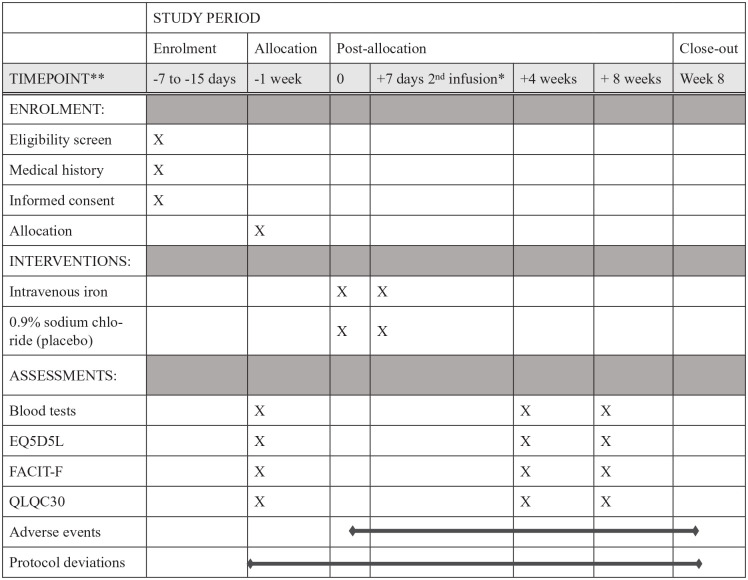
Study schedule. Enrolment, intervention, and outcome measures. *Second infusion if necessary, according to dosing calculation.

All participant blood indices were recorded at baseline and at 4 and 8 weeks follow up visits (Figure 1). These included change in haemoglobin, ferritin, transferrin saturation, serum iron, white cell count, and C-reactive protein. Allogenic blood transfusion events were also recorded. Transferrin saturation and ferritin were used to categorise participants according to absolute or functional iron deficiency (transferrin saturation <20%, ferritin >100 ng/ml) at baseline to inform a subgroup analysis.

Participant quality of life was measured using three validated self-reported questionnaires. These included the Functional Assessment of Cancer Therapy–Fatigue (FACIT-F),^17^ the EuroQol-5D-5 l (EQ-5D-5L)^18^ and the European Organisation for Research and Treatment of Cancer QLQc30 (EORTC QLQc30).^19^ Scoring manuals for these questionnaires are available elsewhere.^20–22^ Several studies describe a minimum clinically important difference (MCID) for FACIT-F scale scores.^23,24^ This was defined for the FACIT-F fatigue scale (3.0 points), the FACIT-General (FACIT-G, 4.0) and the FACIT-F Trial Outcome Index (FACIT-TOI, 5.0). The MCID for the fatigue scale of the QLQC30 was also derived from the available literature at 13 points for improvement and 11.1 for decline (Supplemental Table 3). For the EQ5D5L, simulation-based estimates report a change of 0.063 points as clinically meaningful.^25^ For the visual analogue score (VAS) a MCID of 8–12 points has been reported within a population of people with cancer.^26^

### Sample size

The study sample size was designed to assess feasibility of the trial. It is accepted that a total study sample of 30 participants is sufficient to estimate a study parameter.^27^ Thus, we proposed a target of 40 participants allowing for a 25% dropout rate based on similar trials in this setting.^28^

### Data analysis

All data were analysed on an intention to treat basis. The study was not powered to detect a clinically significant difference in the secondary outcome measures. Statistical analysis was undertaken to explore direction of effect and offer the necessary data to adequately power a definitive trial. Continuous data are presented as mean (standard deviation, SD) for parametric data and median (interquartile range, IQR) for non-parametric data. Values for categorical data are presented as percentages. Paired and unpaired t-tests were used to analyse parametric data. Pearson correlation coefficients were used to assess associations between continuous outcome variables. All tests were two-tailed and statistical significance was defined as *p* < 0.05. Statistical analyses were conducted using SPSS software (Version 27.0. Armonk, NY: IBM Corp). All data were monitored by an independent data monitoring committee.

### Ethical and regulatory approval

The trial was approved by the East Midlands Nottingham 2 Research Ethics Committee (reference 18/EM/0196) and the Health Research Authority. An International Standard Randomised Controlled Trial Number (ISRCTN; reference 13370767) was allocated.

## Results

Thirty-five participants were recruited and 34 were randomised over 16 months from November 2018 to March 2020. Based on to the predicted target of 40 participants in 2 years the recruitment rate progressed ahead of time with 75% of participants being enrolled in the first 12 months. A median of two people were recruited per month (range 0–4). Recruitment in the latter stages of the trial was disrupted due to the COVID-19 pandemic and further accrual was not possible during this time. As the trial had achieved its primary aim of assessing feasibility and had surpassed the 30-participant target, with participant dropout in keeping with our predicted attrition rate, a decision was made to close recruitment at this stage.

## Feasibility

### Screening and recruitment – Are there sufficient participants according to the inclusion criteria who are willing and able to enrol in the proposed study?

**Figure 2.**
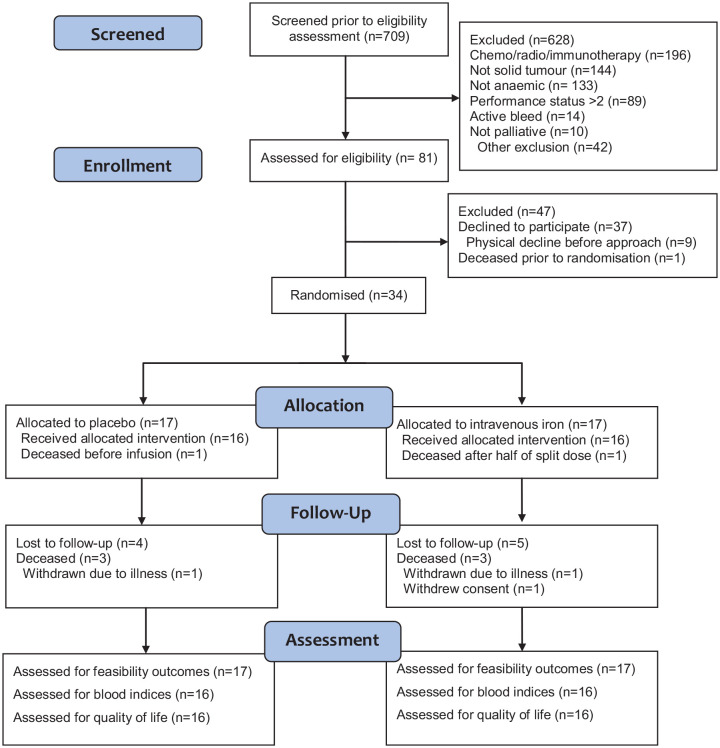
CONSORT diagram for pilot and feasibility trials.

Seven hundred nine people were assessed for eligibility (596 from palliative care clinics or day-therapy, 113 from cancer MDTs). From these, 628 of 709 people were ineligible according to the study exclusion criteria giving a screen failure rate of 89%. After being approached to enrol 47% of people agreed to participate in the study. A reason for decline was not required but many people volunteered their thoughts. Two declined on the grounds of randomisation – and a wish to avoid the placebo arm. One stated that their health insurance would not permit participation in a trial. In all other cases people declined due to the extra burden of visits. None declined due to the chosen outcome measures. Treatment allocation is reported in Figure 2. There were no significant differences in participant baseline characteristics between groups (Table 1). The commonest tumour sites were colorectal (*n* = 6), lung (*n* = 6), oesophageal (*n* = 4) and prostate (*n* = 4).

**Table 1.** Baseline demographics of randomised participants. Mean (SD) or median (IQR [range]) unless specified otherwise. No significant baseline differences existed between groups.

	Placebo (*n* = 17)	Intravenous iron (*n* = 17)
Sex	9F/8M	13M/4F
Age (years)	77 (range 58–84)	74 (range 61–93)
Height (cm)	165 (9.1)	169 (10.1)
Weight (kg)	75.5 (16.9)	73 (17.9)
BMI (kg/m^2^) Haemoglobin (g/L)	26.4 (5.9) 102 (18.1)	25.2 (5.3) 108 (12.5)
Ethnicity		
White British	15	15
Asian Pakistani	1	1
Black Caribbean	1	1
Fatigue score		
(scale range 4–10)	5.6 (1.4)	7 (1.7)
Performance status		
0	1 (6%)	1 (6%)
1	7 (41%)	8 (47%)
2	9 (53%)	8 (47%)
Existing oral iron		
Yes	2 (12%)	1 (6%)
No	15 (88%)	16 (94%)

### Study setting and blinding – What are the logistical and organisational challenges of delivering this trial in the chosen setting?

After enrolment one participant was withdrawn prior to randomisation due to an acute physical deterioration and death. At randomisation five participants (three intravenous iron, two placebo) were newly referred to the palliative care service but had yet to meet a palliative care physician. All other participants were under specialist palliative care services at the point of randomisation. Blinding was maintained during all infusion events.

### Attrition – Are the proposed study duration and follow up schedule appropriate?

Overall participant attrition was 26% (*n* = 9), mostly because of disease progression; this occurred between enrolment to first infusion (*n* = 2), by week 4 (further 5) and week 8 (further 2). One participant from the placebo arm crossed over to the treatment arm after their medical team administered intravenous iron due to worsening anaemia. There were two participants (one in each study arm) who did not attend their 4 weeks follow up appointment due to ill health, but both attended their 8 weeks follow up visit.

### Adherence and protocol deviations – Can the chosen secondary outcome measures be completed effectively and can they inform the primary outcome for a definitive trial?

Among those receiving iron 41% (*n* = 7) of participants received 1000 mg and 59% (*n* = 10) received 1500 mg. Median time from enrolment to infusion was 8 days (range 7–15) and in those requiring a second infusion median duration was 7 days (range 7–14). There were four protocol deviations during the study. Two due to missed study visits and two participants had their infusions delayed by a week. The three quality of life measures appeared acceptable to participants with good rates of questionnaire return (97% baseline, 98% at week 4 and 96% at week 8) and completion (93% of QLQC30, 95% of FACIT-F and 99% of EQ5D5L fully completed).

### Safety – Can intravenous iron be administered safely administered in this group of people?

There were no serious adverse reactions reported during the study. One participant who received iron reported abdominal pain and another experienced headaches. There were no adverse reactions among participants in the placebo arm. During the trial a total of 10 serious adverse events were reported to the independent medical monitor all of which were deemed unrelated to the study intervention (Supplemental Table 4)

#### Haemoglobin and iron studies

**Figure 3.**
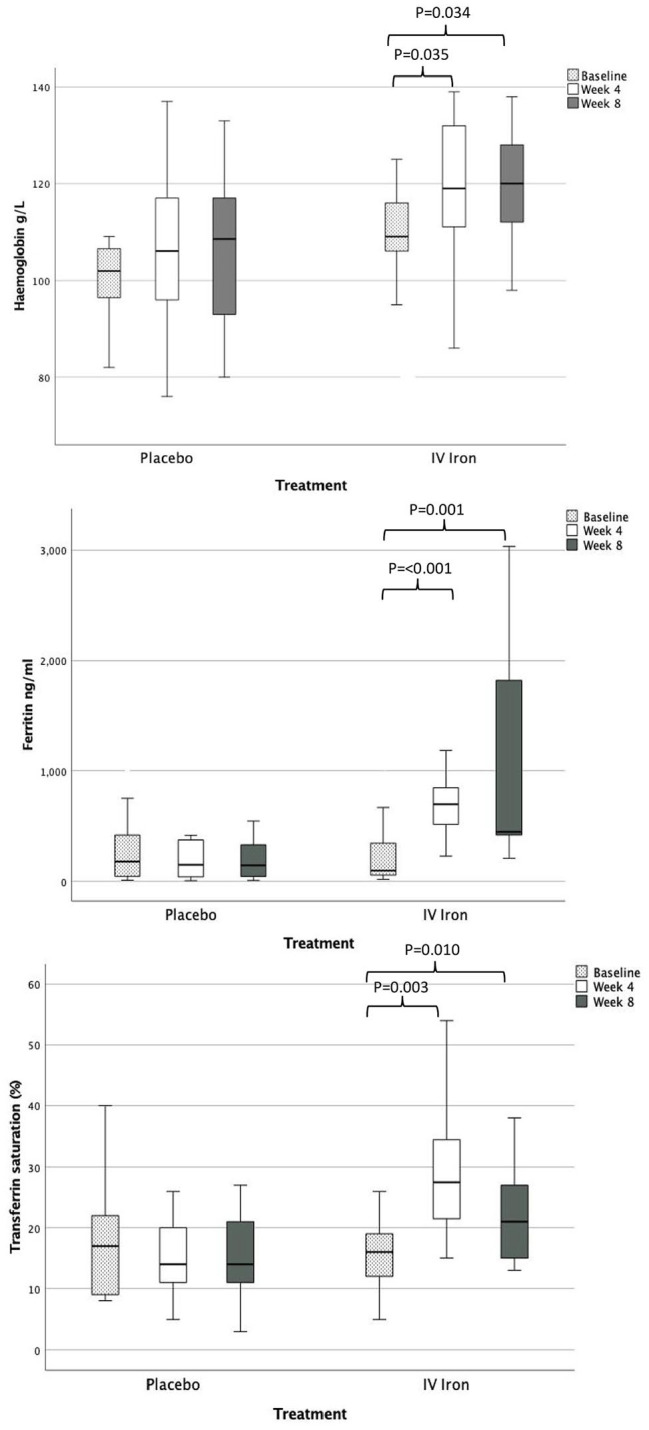
Change in haemoglobin (g/L), ferritin (ng/ml), and transferrin saturation (%) across the three study timepoints for placebo and intravenous iron.

There were no significant differences in haemoglobin or iron studies at baseline between participant groups. Compared to baseline, there was a significant rise in haemoglobin, ferritin, and transferrin saturation % at weeks 4 and 8 for participants in the iron group but not the placebo group (Figure 3). Anaemia resolution was achieved in 39% of intravenous iron participants by week 8 compared to 8% of the placebo group.

A post hoc analysis was performed to categorise participants based on functional, or absolute iron deficiency. When both groups were pooled, the distribution of functional iron deficiency and absolute iron deficiency were similar, with baseline haemoglobin lower in those with functional iron deficiency (functional 102 g/L [SD 17.3], absolute 115 g/L [14]). C-reactive protein was higher for participants with functional iron deficiency than those with absolute iron deficiency, 48 (55) versus 16 (16), respectively. In the iron group, the haemoglobin response was more immediate in participants with absolute iron deficiency compared to functional iron deficiency (Supplemental Figure 1).

#### Transfusion

There were two transfusion events during the study. One occurred in a participant who had an acute drop in haemoglobin and clinical deterioration before randomisation leading to their withdrawal. The other event occurred in a participant in the placebo arm due to an episode of haematemesis related to their oesophageal tumour.

## Quality of life

All scores are reported according to their scoring manuals. The trial was not powered to detect a significant difference in such scores. These data serve to indicate a direction of effect for each study arm.

### Functional Assessment of Cancer Therapy–Fatigue (FACIT-F) scores

There was evidence that intravenous iron may improve fatigue related quality of life for participants according to the FACIT-F fatigue scale. Baseline scores were similar between groups (Table 2) but at both 4 and 8 weeks after infusion the intravenous iron group recorded a clinically important difference of greater than 3.0 points compared to placebo (week 4 mean difference [95% CI] 3.38 [−14.94 to 8.18], week 8 3.49 [−17.52 to 10.53]). A similar improvement was also seen in the FACIT-trial outcome index score (MCID 5.0 points) at 8 weeks for participants in the iron arm. Baseline subscale scores across all FACIT-F domains were also well matched for participants across scales for physical, functional, social and emotional wellbeing (Table 3). There was an indication that intravenous iron may be efficacious at improving participant physical wellbeing at 4 and 8 weeks after infusion. Differences in social, emotional, and functional wellbeing at follow up were less evident between treatment arms.

**Table 2.** FACIT-F derived scores.

Domain	Time	Intravenous iron	Placebo	Mean difference (95% CI)	*p*
FACIT-F Fatigue Scale [maximum 52]	Baseline	22.6 (17.9)	22.2 (13.3)	0.39 (−14.31-13.55)	0.955
	Week 4	28.4 (13.4)	25 (12.5)	3.38* (−14.94-8.18)	0.548
	Week 8	24.8 (17.2)	21.3 (14.4)	3.49* (−17.52-10.53)	0.609
Trial Outcome Index [maximum 108]	Baseline	53 (30.1)	53.6 (22.1)	−0.65 (−22.52-23.82)	0.953
	Week 4	58.8 (26.4)	55.4 (20.1)	3.45 (−24.13-17.24)	0.731
	Week 8	59.2 (29.8)	52.9 (22.1)	6.28* (−29.34-16.78)	0.576
FACIT General [maximum 108]	Baseline	67.3 (23.4)	68.2 (17.6)	−0.83 (−17.38-19.05)	0.925
	Week 4	72.4 (23.4)	69.2 (11.9)	3.27 (−19.35-12.81)	0.676
	Week 8	73.2 (23.6)	70.3 (13.5)	2.89 (−19.61-13.82	0.721
FACIT-F Total [maximum 160]	Baseline	89.9 (37.8)	90.3 (27.4)	−0.45 (−28.60-29.51)	0.974
	Week 4	97.5 (36)	95 (21.3)	2.5 (−28.3-23.3)	0.840
	Week 8	98 (39.3)	91.6 (24.6)	6.4 (-35–22.3)	0.647

Values are mean (SD) or with 95% confidence interval as indicated. The higher the score, the better the quality of life.

* Denotes minimum clinically important difference exceeded between groups.

**Table 3.** FACIT-F subscale domains.

Domain	Time	Intravenous iron	Placebo	Mean difference (95% CI)	*P*
Physical Wellbeing [maximum 28]	Baseline	16.3 (8.5)	16.6 (5.5)	−0.28 (−5.96–6.53)	0.925
	Week 4	18.8 (8.6)	16.1 (4.9)	2.74 (−8.81-3.32)	0.357
	Week 8	19.6 (7.4)	16.0 (6.4)	3.57 (−9.71-2.56)	0.238
Social Wellbeing [maximum 28]	Baseline	20.6 (7)	21.4 (6.6)	−0.8 (−5.26-6.85)	0.786
	Week 4	21.1 (6.9)	22.3 (4.9)	−1.2 (−4.06-6.45)	0.639
	Week 8	21.3 (6.5)	22.5 (4.9)	−1.14 (−3.95-6.22)	0.645
Emotional Wellbeing [maximum 24]	Baseline	16.4 (6.1)	15.3 (5)	0.99 (−5.9-3.91)	0.677
	Week 4	16.5 (4.4)	17.6 (5)	1.1 (−5.28-3.09)	0.589
	Week 8	17.5 (5.9)	16.2 (4.6)	1.24 (−5.92-3.4)	0.585
Functional Wellbeing [maximum 28]	Baseline	14.1 (6)	14.8 (6.4)	−0.75 (−4.81-6.31)	0.781
	Week 4	14.9 (6.2)	14.3 (4.8)	0.62 (−5.54-4.29)	0.794
	Week 8	14.8 (7.3)	15.6 (5.2)	−0.78 (−4.8-6.37)	0.772

Values are mean (SD) or with 95% confidence interval as indicated. The higher the score, the greater the wellbeing.

### QLQC30 scores

Baseline global health scores were lower for participants in the iron group but not significantly so. At 4 weeks, compared to baseline, there was an improvement in mean global health scores for those in both the iron and placebo groups (iron mean difference (95% CI) 10.8 [−2 to 23.7] *p* = 0.089, placebo 4.8 [−8 to 17.7], *p* = 0.423). No significant inter or intragroup differences were seen in scores at 8 weeks follow up (Supplemental Figure 3). There was also evidence to indicate that participant quality of life related to physical, emotional and social functioning improved after intravenous iron, whereas in the placebo arm physical functioning scores decreased (Supplemental Table 6).

### EQ5D5L scores

Self reported VAS scores were higher for the participants in the placebo arm at baseline. However, at both 4 and 8 weeks the iron group saw an increase in VAS score whereas the placebo group saw scores reduce at both follow up timepoints (Supplemental Figure 4). At 4 weeks, compared to baseline, index scores for present health state increased for participants in both groups by a clinically important difference (0.063 points) at 4 weeks, but only the iron group saw these improvements last to 8 weeks.

### Correlation between quality of life and haemoglobin

To determine whether an increase in haemoglobin and haematinic markers were associated with an improvement in quality of life Pearson correlation coefficients were plotted between haemoglobin and all major components of the three questionnaires. A significant positive correlation was seen for the FACIT-G score (*r* = .257, *p* = 0.021) and the global health component of the QLQC30 questionnaire (*r* = 0.348, *p* = 0.009). Transferrin saturation % was also positively correlated with the FACIT-G (*r* = 0.245, *p* = 0.042) across all three study timepoints.

## Discussion

### Main findings

The ICaRAS trial has demonstrated that intravenous iron administration was feasible and well tolerated in people with advanced cancer being managed in a palliative setting according to recruitment and attrition rates. The chosen research questions were all assessed via the feasibility outcomes and the results of the trial now act as a guide for the design of a definitive study. The direction of effect in our secondary outcome measures suggests that intravenous iron may be an efficacious means of improving haemoglobin compared to placebo in this group of people. These changes were sustained until at least 8 weeks after treatment and anaemia resolution was achieved in a higher proportion of participants in the intravenous iron group. We also found that changes in ferritin and transferrin saturation follow similar trends to those seen in the literature^29^ and indicate that a single dose of ferric derisomaltose may be efficacious at replacing iron stores in these individuals.

The three quality of life questionnaires were acceptable and feasible within the present study design and the successful blinding of participants to their study allocation further adds validity. Despite our small sample size there was a signal that threshold of the minimum clinically important difference across several quality of life measures was passed for participants in the iron group. Namely, the fatigue scales of both the FACIT-F and QLQC30 as well as the FACIT-F Trial Outcome Index.

It was not possible to draw any meaningful conclusions on the impact of intravenous iron on the frequency of blood transfusion in this group of people, but it is encouraging that no transfusion events occurred in the iron group. Further data are needed to examine this observation.

### What this study adds

The study has demonstrated that it is feasible to recruit people to a randomised controlled trial of intravenous iron according to our enrolment of 1–4 participants per month. The participant attrition rate was also in keeping with the rate proposed in our protocol at 25%. Further, the feasibility target of 30 participants was also passed^27^ and our acceptance rate was 47% indicating that around half of people approached agreed with the prospect of randomisation. These findings are encouraging given that similar trials in advanced cancer usually experience participant dropout in the region of 22%^30^ but can be as high as 60% or more^31^ particularly in studies of fatigue.^28^ Median attrition rates due to participant death before study completion have been reported to be in the region of 8% of all cases of dropout in other studies.^28^

Changes seen in haemoglobin with iron offer an important objective measures through which clinicians might evaluate treatment response among recipients and will guide future trial design. Similar, retrospective, uncontrolled studies of iron therapy in people with iron deficiency receiving palliative care have reported higher haemoglobin increases of 15.1–18.7 g/L over 4–8 weeks following iron infusion^32^ Such studies also recruited participants with a lower baseline haemoglobin to those in ICaRAS. Importantly, there is also a degree of both laboratory and physiologic that is diurnal variation in haemoglobin.^33^ Although these factors would affect participants in both study arms their impact may be amplified in a small feasibility study.

A positive correlation was seen between haemoglobin rise and selected quality of life domains such as the FACT-G derived score. This highlights an area for further evaluation given that previous studies suggest that only a weak correlation might exist.^34^ Participant haemoglobin response according to classification of iron deficiency (absolute or functional) also generated some interesting observations. We found haemoglobin response appeared to occur later in people with functional iron deficiency indicating that ferric derisomaltose may be more efficacious at correcting absolute iron deficiency. It must be stressed that this analysis was undertaken on a subgroup of study participants and as such should be interpreted with caution. Regardless, this observation has not been reported in the literature and further exploration may guide treatment response or repeat dosing in a clinical setting.

Quality of life reporting should be seen as the most important participant centred outcome, and this is likely to represent the most meaningful primary outcome measure in a definitive trial. ICaRAS was not powered to detect clinically meaningful changes in quality of life but there is an indication that intravenous iron may offer favourable improvements for participants compared to placebo for the FACIT-F fatigue scale. The FACIT-F has been widely studied among similar participant populations and has been used as a primary outcome measure in contemporary trials of treatments for fatigue in people with advanced cancer^35^ allowing a degree of comparison between intravenous iron and other interventions. Median baseline FACIT-F fatigue scores for all participants (placebo 22.2, intravenous iron 22.6) were also in keeping with those quoted in the literature (23.9) for anaemic, fatigued people with cancer^36^ demonstrating that ICaRAS has correctly targetted the population of interest.

The observations seen in fatigue scale scores for placebo group participants indicated an increase in fatigue over the 8 weeks. These data support the understanding of a functional decline in people with advanced cancer, particularly in the last year of life.^37^ This may also offer support for intravenous iron to prevent or delay increasing fatigue associated with disease progression. Importantly, several quality of life measures improved for placebo group participants during follow up. These improvements may reflect the multidisciplinary palliative care support being received outside of the trial setting with subsequent improvements in psychological and physical wellbeing. We also hypothesise that improvements in placebo group participants may have occurred due to the ‘Hawthorne effect’^38^ which can still arise despite concealing treatment allocation.^39^ Similar results have been seen in other placebo-controlled trials studying fatigue in people with advanced cancer, even with the use of blinding.^40,41^ To counter this effect, we may have to eliminate placebo responders during a run-in phase of any future clinical trial.^42^

### Study limitations

The secondary outcome measures are intended to indicate a direction of effect and all such results should be interpreted with caution in this unpowered study. Participant recruitment and attrition data are encouraging but may differ outside of a specialist palliative service within secondary care. The disruption of the COVID-19 pandemic also impacted final recruitment figures. Despite this there were important indicators of efficacy for intravenous iron across both objective and subjective outcome measures and our recruitment threshold of 30 participants was passed.^27^

In conclusion, the present study design was feasible and demonstrated that intravenous iron may be efficacious at improving haemoglobin and iron stores in people with palliative cancer suffering from symptomatic anaemia. These changes appeared to be linked to improvements in participant quality of life across select measures including fatigue-specific domains. The results should inform the design of a definitive trial of this treatment for people with anaemia in advanced cancer.

## Supplemental Material

sj-pdf-1-pmj-10.1177_02692163221145604 – Supplemental material for The ICaRAS randomised controlled trial: Intravenous iron to treat anaemia in people with advanced cancer – feasibility of recruitment, intervention and delivery Click here for additional data file. Supplemental material, sj-pdf-1-pmj-10.1177_02692163221145604 for The ICaRAS randomised controlled trial: Intravenous iron to treat anaemia in people with advanced cancer – feasibility of recruitment, intervention and delivery by Edward A Dickson, Oliver Ng, Barrie D Keeler, Andrew Wilcock, Matthew J Brookes and Austin G Acheson in Palliative Medicine

## References

[1] Dunn A Carter J Carter H . Anemia at the end of life: prevalence, significance, and causes in patients receiving palliative care. J Pain Symptom Manag 2003; 26(6): 1132–1139. 10.1016/j.jpainsymman.2003.04.001 14654265

[2] Cella D Kallich J McDermott A , et al. The longitudinal relationship of hemoglobin, fatigue and quality of life in anemic cancer patients: results from five randomized clinical trials. Ann Oncol 2004; 15(6): 979–986. 15151958 10.1093/annonc/mdh235

[3] Neoh K Gray R Grant-Casey J , et al. National comparative audit of red blood cell transfusion practice in hospices: recommendations for palliative care practice. Palliat Med 2019; 33(1): 102–108. 30260291 10.1177/0269216318801755 PMC6291900

[4] Mercadante S Ferrera P Villari P , et al. Effects of red blood cell transfusion on anemia-related symptoms in patients with cancer. J Palliat Med 2009; 12(1): 60–63. 19284264 10.1089/jpm.2008.0139

[5] Bohlius J Schmidlin K Brillant C , et al. Recombinant human erythropoiesis-stimulating agents and mortality in patients with cancer: a meta-analysis of randomised trials. Lancet 2009; 373(9674): 1532–1542. 19410717 10.1016/S0140-6736(09)60502-X

[6] Vansteenkiste J Glaspy J Henry D , et al. Benefits and risks of using erythropoiesis-stimulating agents (ESAs) in lung cancer patients: study-level and patient-level meta-analyses. Lung Cancer 2012; 76(3): 478–485. 22277104 10.1016/j.lungcan.2011.12.015

[7] Tolkien Z Stecher L Mander AP , et al. Ferrous sulfate supplementation causes significant gastrointestinal side-effects in adults: a systematic review and meta-analysis. PLoS One 2015; 10(2): e0117383. 10.1371/journal.pone.0117383 PMC4336293 25700159

[8] Gereklioglu C Asma S Korur A , et al. Medication adherence to oral iron therapy in patients with iron deficiency anemia. Pak J Med Sci 2016; 32(3): 604–607. 27375698 10.12669/pjms.323.9799 PMC4928407

[9] Stoffel NU Cercamondi CI Brittenham G , et al. Iron absorption from oral iron supplements given on consecutive versus alternate days and as single morning doses versus twice-daily split dosing in iron-depleted women: two open-label, randomised controlled trials. Lancet Haematol 2017; 4(11): e524–e33. 10.1016/S2352-3026(17)30182-5 29032957

[10] Ganz T . Hepcidin—a regulator of intestinal iron absorption and iron recycling by macrophages. Best Pract Res Clin Haematol 2005; 18(2): 171–182. 15737883 10.1016/j.beha.2004.08.020

[11] Bregman DB Morris D Koch TA , et al. Hepcidin levels predict nonresponsiveness to oral iron therapy in patients with iron deficiency anemia. Am J Hematol 2013; 88(2): 97–101. 23335357 10.1002/ajh.23354

[12] Keeler BD Simpson JA Ng O , et al. Randomized clinical trial of preoperative oral versus intravenous iron in anaemic patients with colorectal cancer. Br J Surg 2017; 104(3): 214–221. 28092401 10.1002/bjs.10328

[13] Kulnigg S Stoinov S Simanenkov V , et al. A novel intravenous iron formulation for treatment of anemia in inflammatory bowel disease: the ferric carboxymaltose (FERINJECT) randomized controlled trial. Am J Gastroenterol 2008; 103(5): 1182–1192. 18371137 10.1111/j.1572-0241.2007.01744.x

[14] Anker SD Comin Colet J Filippatos G , et al. Ferric carboxymaltose in patients with heart failure and iron deficiency. New Engl J Med 2009; 361(25): 2436–2448. 19920054 10.1056/NEJMoa0908355

[15] Qunibi WY Martinez C Smith M , et al. A randomized controlled trial comparing intravenous ferric carboxymaltose with oral iron for treatment of iron deficiency anaemia of non-dialysis-dependent chronic kidney disease patients. Nephrol Dial Transplant 2011; 26(5): 1599–1607. 20929915 10.1093/ndt/gfq613 PMC3084440

[16] Avni T Bieber A Grossman A , et al. The safety of intravenous iron preparations: systematic review and meta-analysis. Mayo Clin Proc 2015; 90(1): 12–23. 25572192 10.1016/j.mayocp.2014.10.007

[17] Yellen SB Cella DF Webster K , et al. Measuring fatigue and other anemia-related symptoms with the functional assessment of cancer therapy (FACT) measurement system. J Pain Symptom Manag 1997; 13(2): 63–74. 10.1016/s0885-3924(96)00274-6 9095563

[18] Devlin NJ Shah KK Feng Y , et al. Valuing health-related quality of life: an EQ-5D-5L value set for England. Health Econ 2018; 27(1): 7–22. 28833869 10.1002/hec.3564 PMC6680214

[19] Aaronson NK Ahmedzai S Bergman B , et al. The European Organization for research and Treatment of Cancer QLQ-C30: a quality-of-life instrument for use in international clinical trials in oncology. J Natl Cancer Inst 1993; 85(5): 365–376. 8433390 10.1093/jnci/85.5.365

[20] FACIT. Functional Assessment of Chronic Illness Therapy – Fatigue Scale scoring manual, https://www.facit.org/measures/FACIT-Fatigue

[21] Cancer EOfRaTo. The EORTC QLQ-C30 scoring manual 2021, https://www.eortc.org/app/uploads/sites/2/2018/02/SCmanual.pdf

[22] Foundation ER . EurolQol EQ5D5L scoring manual 2021, https://euroqol.org/publications/user-guides/

[23] Reddy S Bruera E Pace E , et al. Clinically important improvement in the intensity of fatigue in patients with advanced cancer. J Palliat Med 2007; 10(5): 1068–1075. 17985963 10.1089/jpm.2007.0007

[24] Patrick DL Gagnon DD Zagari MJ , et al. Assessing the clinical significance of health-related quality of life (HrQOL) improvements in anaemic cancer patients receiving epoetin alfa. Eur J Cancer 2003; 39(3): 335–345. 12565986 10.1016/s0959-8049(02)00628-7

[25] McClure NS Sayah FA Xie F , et al. Instrument-defined estimates of the minimally important difference for EQ-5D-5L Index scores. Value Health 2017; 20(4): 644–650. 28408007 10.1016/j.jval.2016.11.015

[26] Pickard AS De Leon MC Kohlmann T , et al. Psychometric comparison of the standard EQ-5D to a 5 level version in cancer patients. Med Care 2007; 45(3): 259–263. 17304084 10.1097/01.mlr.0000254515.63841.81

[27] Lancaster GA Dodd S Williamson PR . Design and analysis of pilot studies: recommendations for good practice. J Eval Clin Pract 2004; 10(2): 307–312. 15189396 10.1111/j..2002.384.doc.x

[28] Hui D Glitza I Chisholm G , et al. Attrition rates, reasons, and predictive factors in supportive care and palliative oncology clinical trials. Cancer 2013; 119(5): 1098–1105. 23132290 10.1002/cncr.27854 PMC3568443

[29] Ng O Keeler B Simpson JA , et al. Feasibility of intravenous iron isomaltoside to improve anemia and quality of life during palliative chemotherapy for esophagogastric adenocarcinoma. Nutr Cancer 2018; 70(7): 1106–1117. 30198775 10.1080/01635581.2018.1504090

[30] Bouça-Machado R Rosário M Alarcão J , et al. Clinical trials in palliative care: a systematic review of their methodological characteristics and of the quality of their reporting. BMC Palliat Care 2017; 16(1): 10. 28122560 10.1186/s12904-016-0181-9 PMC5264484

[31] Addington-Hall JM MacDonald LD Anderson HR , et al. Randomised controlled trial of effects of coordinating care for terminally ill cancer patients. BMJ 1992; 305(6865): 1317–1322. 1483075 10.1136/bmj.305.6865.1317 PMC1883850

[32] Steele T Bonwick H Nwosu AC , et al. Investigation and management of iron deficiency anaemia in a specialist palliative care setting and the role of intravenous iron: a descriptive analysis of hospice data. AMRC Open Research 2021; 3(6): 6. 10.12688/amrcopenres.12963.2 PMC11064982 38708071

[33] Pocock SJ Ashby D Shaper AG , et al. Diurnal variations in serum biochemical and haematological measurements. J Clin Pathol 1989; 42(2): 172–179. 2921359 10.1136/jcp.42.2.172 PMC1141821

[34] Munch TN Zhang T Willey J , et al. The association between anemia and fatigue in patients with advanced cancer receiving palliative care. J Palliat Med 2005; 8(6): 1144–1149. 16351527 10.1089/jpm.2005.8.1144

[35] Stone P King M Richardson A , et al. MePFAC: Methylphenidate versus placebo for fatigue in advanced cancer 2020, https://www.ucl.ac.uk/psychiatry/research/marie-curie-palliative-care-research/research/supportive-and-end-of-life-care/mepfac 10.3310/GJPS6321 PMC12376206 40755226

[36] Cella D Lai JS Chang CH , et al. Fatigue in cancer patients compared with fatigue in the general United States population. Cancer 2002; 94(2): 528–538. 11900238 10.1002/cncr.10245

[37] Lunney JR Lynn J Foley DJ , et al. Patterns of functional decline at the end of life. JAMA 2003; 289(18): 2387–2392. 12746362 10.1001/jama.289.18.2387

[38] McCarney R Warner J Iliffe S , et al. The Hawthorne effect: a randomised, controlled trial. BMC Med Res Methodol 2007; 7(1): 30. 17608932 10.1186/1471-2288-7-30 PMC1936999

[39] Sedgwick P Greenwood N . Understanding the Hawthorne effect. BMJ: British Medical Journal 2015; 351: h4672. 10.1136/bmj.h4672 26341898

[40] Centeno C Rojí R Portela MA , et al. Improved cancer-related fatigue in a randomised clinical trial: methylphenidate no better than placebo. BMJ Support Palliat Care 2022; 12: 226–234. 10.1136/bmjspcare-2020-002454 33168668

[41] Bruera E Valero V Driver L , et al. Patient-controlled methylphenidate for cancer fatigue: a double-blind, randomized, placebo-controlled trial. J Clin Oncol 2006; 24(13): 2073–2078. 16648508 10.1200/JCO.2005.02.8506

[42] Muthén B Brown HC . Estimating drug effects in the presence of placebo response: causal inference using growth mixture modeling. Stat Med 2009; 28(27): 3363–3385. 19731223 10.1002/sim.3721 PMC2818509

