# Supplementary material for: The ICaRAS randomised controlled trial: Intravenous iron to treat anaemia in people with advanced cancer – feasibility of recruitment, intervention and delivery
Source: Palliat Med. 2023 Jan 6;37(3):372–83. doi: 10.1177/02692163221145604 (PMC10021128; doi:10.1177/02692163221145604)
Supplement: sj-pdf-1-pmj-10.1177_02692163221145604 – Supplemental material for The ICaRAS randomised controlled trial: Intravenous iron to treat anaemia in people with advanced cancer – feasibility of recruitment, intervention and delivery [file sj-pdf-1-pmj-10.1177_02692163221145604.pdf]

Supplementary table 1 – Participant inclusion and exclusion criteria for ICaRAS

**Inclusion Criterion**
Able to consent to participate?
Willing to allow GP/Consultant to be informed of participation in study?
Willing / able to participate with all aspects of the study?
Anaemic (<130g/L Male, <120 g/L Female) on latest full blood count within 2 months.
Cancer not amenable to curative treatment?
Fatigue numeric rating scale score ≥ 4 out of 10
Histologically or radiologically proven solid tumour?
ECOG Performance status 0-2
**Exclusion Criterion**
Evidence of iron overload or disturbance of iron utilisation or iron infusion in the past 3 months
Evidence of allergy to iron or related product
Pregnant, lactating or planning a pregnancy during the study
Evidence of untreated infection
Evidence of thromboembolic event (DVT, PE) within 3 months unless ongoing treatment with anticoagulation
Untreated haematological malignancy?
Evidence of active bleeding
Undergoing concurrent anti-cancer chemotherapy and/or immunotherapy and/or radiotherapy (within 8 weeks)
Unwilling to stop any existing oral iron supplementation

Supplementary table 2 - Simplified dosing regimen for Iron III Isomaltoside. For people with a BMI >30 an ideal body weight was used.

Hb (g/L)	Weight 50 kg to<70 kg	Weight ≥70 kg
≥100	1000 mg	1500 mg
<100	1500 mg	2000 mg

Hb, haemoglobin; mg, milligrams.

Supplementary Table 3 - Studies reporting anchor-based a QLQC30 Fatigue Scale minimum clinically important difference (MCID)

Authors and participant diagnosis	MCID Improvement	MCID Decline
Bedard et al. 2014<br>Cancer (n = 369)	13.6–17.3	Not stated
Kvam et al. 2010<br>Multiple myeloma<br>(n = 239)	13.5	8.6
Bedard et al. 2013b<br>Cancer (n = 276)	Not stated	24.5
Maringwa et al. 2011a<br>Brain cancer<br>(n = 941)	12.4	8.9
Maringwa et al. 2011b<br>Lung cancer (n = 812)	14.1	5.7
Zeng et al. 2012<br>Cancer (n = 93)	11.4	7.8

Supplementary Table 4 - Serious adverse events (SAE) reported during the ICaRAS trial.

**System organ class (SOC)**<br>**- Lower-level term (LLT)**	**Number of SAEs**
Infections and Infestations<br>- Pneumonia	1 (Intravenous iron)
Cardiac disorders<br>- Myocardial Infarction	1 (Intravenous iron)
Neoplasms benign, malignant and unspecified<br>- Brain metastasis secondary to ovarian cancer	1 (Intravenous iron)
Blood and Lymphatic System Disorders<br>- Anaemia requiring blood transfusion	1 (placebo)
Gastrointestinal Disorders<br>- Diarrhoea and vomiting	1 (placebo)
Skin and subcutaneous tissue disorders<br>- Leg oedema and cellulitis<br>- Pressure sore	2 (placebo)
Musculoskeletal and connective tissue disorders<br>- Exacerbation of chronic back pain requiring inpatient admission<br>- Exacerbation of chronic leg pain requiring inpatient admission	2 (placebo)
Surgical and medical procedures<br>- Insertion of oesophageal stent	1 (Intravenous iron)

Supplementary table 5 - Change in participant haemoglobin and iron studies across follow up. Values shown are mean (SD). P values relate to intragroup change from baseline.

		**Baseline**	**Week 4**	**Week 8**
**Placebo**	**Haemoglobin** g/L	102 (18.1)	106 (17.9) P=0.418	107 (15.8) P=0.848
	**Iron** mmol/L	8.57 (4.7)	6.7 (2.8) P=0.153	8.1 (3.8) P=0.715
	**Ferritin** ng/ml	201 (220)	243 (294) P=0.636	209 (197) P=0.947
	**TSAT** %	16.5 (8.6)	14.8 (6) P=0.602	18.4 (10.3) P=0.310
**Intravenous iron**	**Haemoglobin** g/L	108 (12.5)	117 (17.5) P=0.035	118.1 (13.9) P=0.034
	**Iron** mmol/L	10.2 (3.2)	14.1 (6.2) P=0.015	11 (6.1) P=0.560
	**Ferritin** ng/ml	211 (283)	908 (737) P=<0.001	996 (907) P=0.001
	**TSAT** %	16.8 (4.5)	31.2 (12.2) P=0.003	22.6 (8.2) P=0.010

Supplementary Table 6 – Participant QLQC30 functional scores. Values are presented as mean (SD). Mean difference is presented with. 95% CI in parentheses. Maximum total score for each domain = 100. Higher scores reflect higher function.

Domain	Time	Intravenous iron	Placebo	*Mean difference*	*P*
**Physical Functioning**	Baseline	44.4 (30.5)	45.4 (21.3)	-1 (-21.9-24.4)	0.912
	Week 4	53.3 (30.6)	38.6 (19.9)	14.7 (-37.3-7.9)	0.189
	Week 8	42.7 (29.8)	38.9 (22.5)	3.8 (-27.1-19.5)	0.738
**Role Functioning**	Baseline	40 (31.6)	50 (22.5)	-10 (-14.1-34.1)	0.396
	Week 4	48.3 (34.6)	30.6 (18.6)	17.8 (-41.9-6.4)	0.140
	Week 8	41.7 (31.7)	48.6 (28.8)	-6.9 (-20-33.9)	0.596
**Emotional Functioning**	Baseline	59.2 (32)	63.7 (23.7)	-4.5 (-20.3-29.3)	0.709
	Week 4	78.3 (29.5)	67.8 (25.4)	10.5 (-34.9-13.9)	0.378
	Week 8	74.2 (33)	63.9 (23.7)	10.3 (-35.5-14.9)	0.405
**Cognitive Functioning**	Baseline	53.3 (30.2)	59.7 (27)	-6.4 (-19.1-31.8)	0.606
	Week 4	56.7 (29.6)	61.1 (28.7)	-4.4 (-21.6-30.5)	0.725
	Week 8	66.7 (30.4)	55.6 (28.7)	11.1 (-37.4-15.3)	0.390
**Social Functioning**	Baseline	43.3 (40.2)	52.8 (34.7)	-9.4 (-23.9-42.7)	0.560
	Week 4	56.7 (37.8)	52.8 (24.4)	3.9 (-31.8-24)	0.773
	Week 8	58.3 (33.6)	47.2 (34.7)	11.1 (-41.6-19.4)	0.457

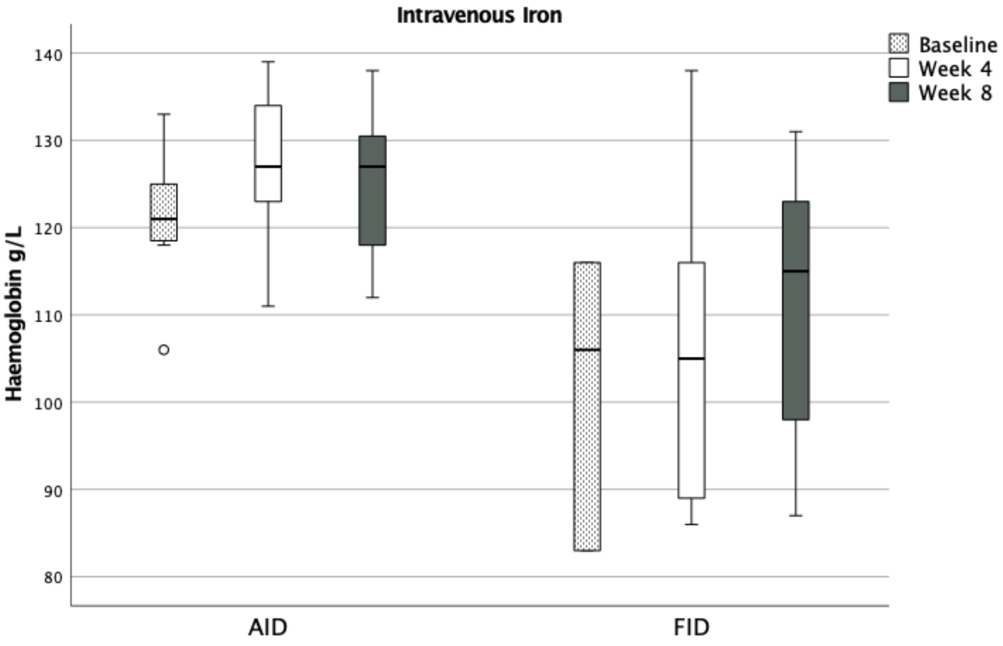

Supplementary Figure 1 - Change in haemoglobin for participants receiving intravenous iron according to baseline iron deficiency anaemia status. AID, absolute iron deficiency; FID, functional iron deficiency

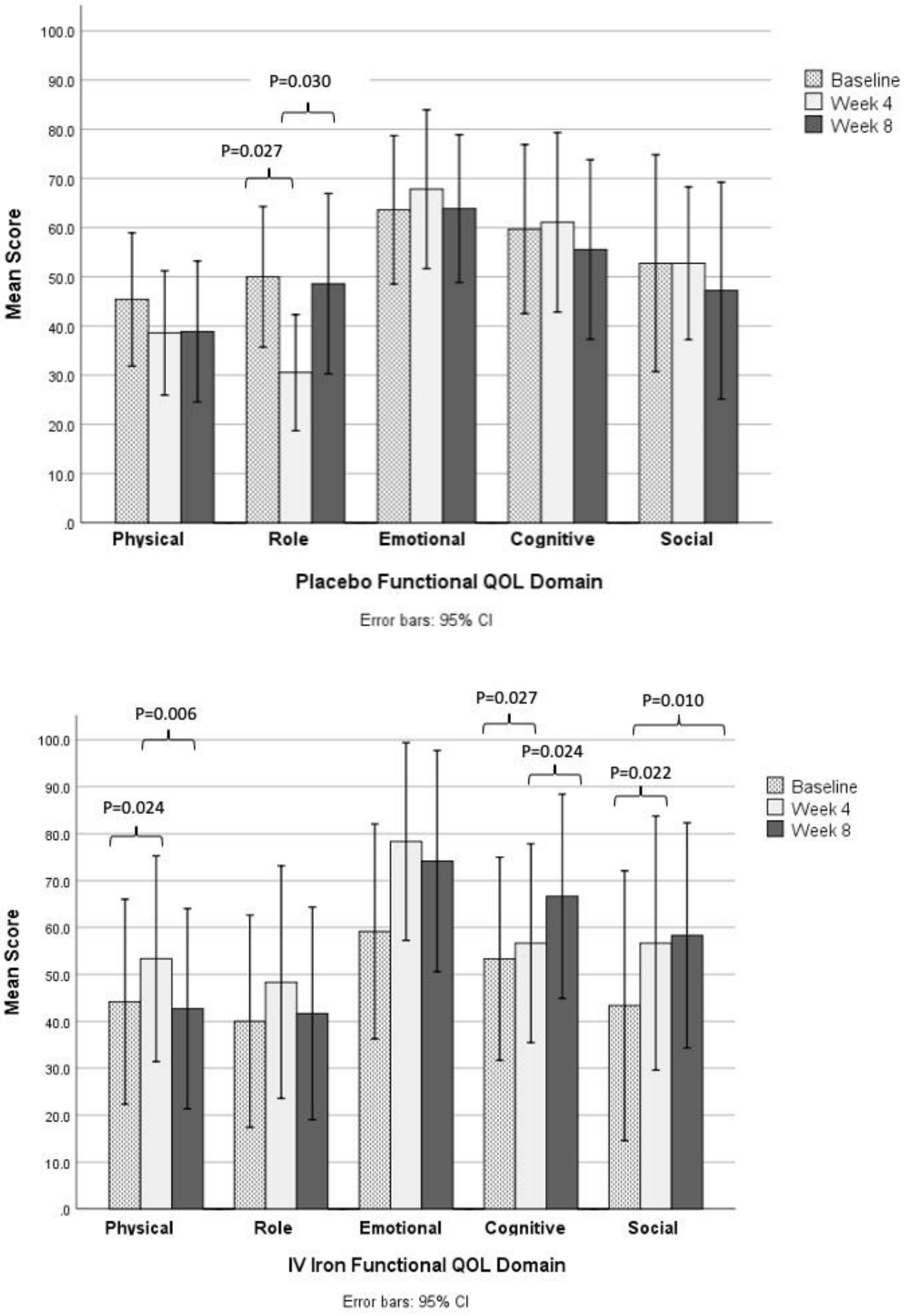

Supplementary figure 2- Mean functional domain scores of the QLQC30 questionnaire for placebo and intravenous iron groups at the 3 study timepoints. P values denote statistically significant intragroup changes between timepoints.

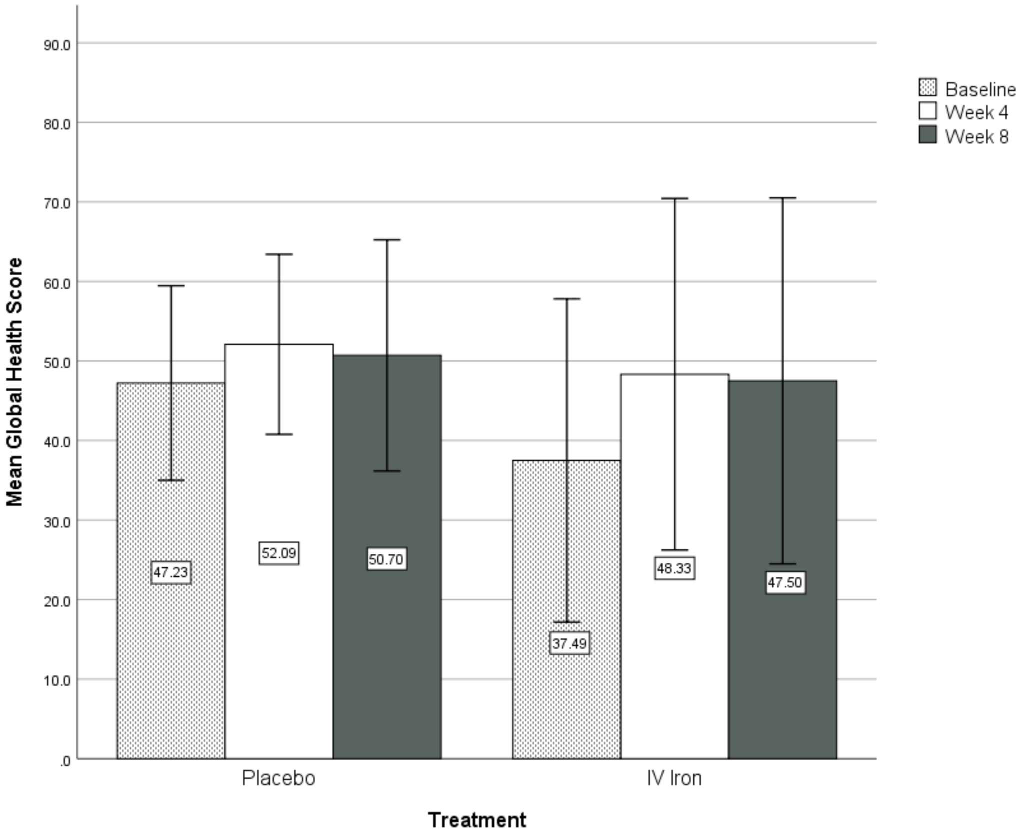

Supplementary figure 3 - Mean QLQC30 global health scores for participants in the placebo and intravenous iron group at the 3 study timepoints. Higher scores represent a better quality of life

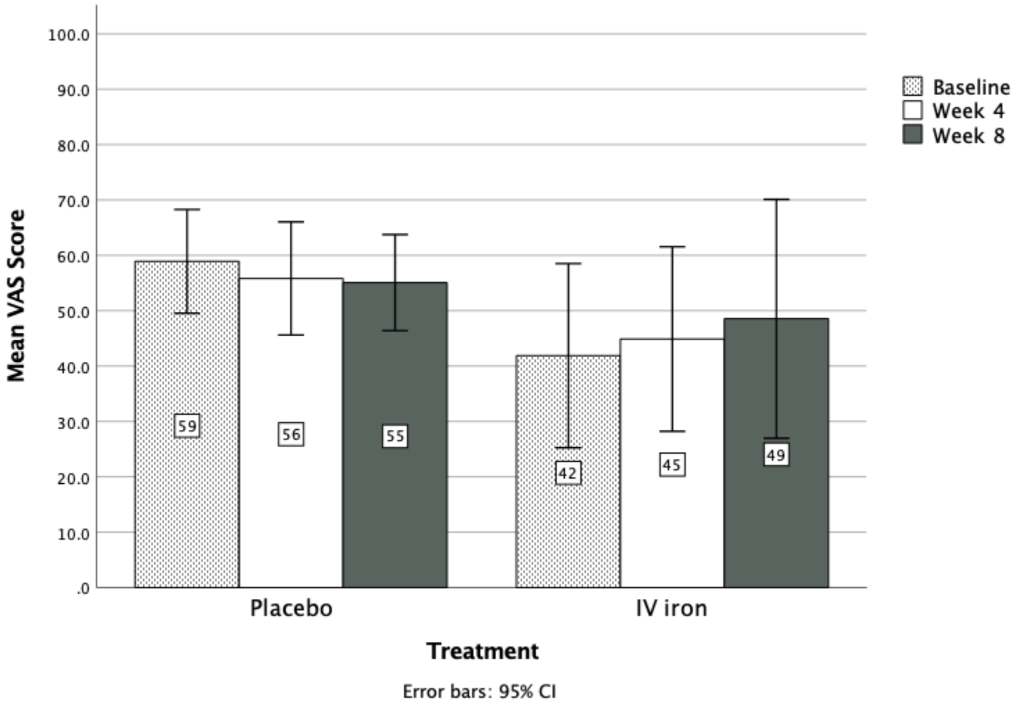

Supplementary figure 4 - Mean visual analogue scale (VAS) scores for participants in the placebo and Intravenous iron groups. Scores represent a persons perceived health state on the day of reporting. Scores are recorded on a scale of 0-100 (0, worst health imaginable; 100, best health imaginable)
